# MMPP Exerts Anti-Inflammatory Effects by Suppressing MD2-Dependent NF-κB and JNK/AP-1 Pathways in THP-1 Monocytes

**DOI:** 10.3390/ph16040480

**Published:** 2023-03-23

**Authors:** Seonhwa Kim, Na-Yeon Kim, Jae-Young Park, Hyo-Min Park, Chae-Min Lim, Jinju Kim, Hee Pom Lee, Jin Tae Hong, Do-Young Yoon

**Affiliations:** 1 Department of Bioscience and Biotechnology, Konkuk University, Seoul 05029, Republic of Korea; 2 College of Pharmacy and Medical Research Center, Chungbuk National University, Cheongju 28160, Republic of Korea

**Keywords:** MMPP, lipopolysaccharide, MD2/TLR4, NF-κB, AP-1

## Abstract

(E)-2-methoxy-4-[3-(4-methoxyphenyl) prop-1-en-1-yl] phenol (MMPP), a novel synthetic analog of (E)-2,4-bis(p-hydroxyphenyl)-2-butenal (BHPB), exerts anti-inflammatory and anticancer effects by downregulating the STAT3 pathway. It has also been recently reported that MMPP can act as a PPAR agonist which enhances glucose uptake and increases insulin sensitivity. However, it has not yet been elucidated whether MMPP can act as an antagonist of MD2 and inhibit MD2-dependent pathways. In this study, we evaluated the underlying modulatory effect of MMPP on inflammatory responses in LPS-stimulated THP-1 monocytes. MMPP inhibited the LPS-induced expression of inflammatory cytokines, such as TNF-α, IL-1β, and IL-6, as well as the inflammatory mediator COX-2. MMPP also alleviated the IKKαβ/IκBα and JNK pathways and the nuclear translocation of NF-κB p50 and c-Jun in LPS-stimulated THP-1 monocytes. In addition, the molecular docking analyses and in vitro binding assay revealed that MMPP can directly bind to CD14 and MD2, which are expressed in the plasma membrane, to recognize LPS first. Collectively, MMPP was directly bound to CD14 and MD2 and inhibited the activation of the NF-κB and JNK/AP-1 pathways, which then exerted anti-inflammatory activity. Accordingly, MMPP may be a candidate MD2 inhibitor targeting TLR4, which exerts anti-inflammatory effects.

## 1. Introduction

Monocytes are one type of innate immune cells which is developed in the bone marrow [1]. They constantly circulate through the bloodstream and pass into tissues in response to homeostasis and inflammatory signals [2]. Under the action of lipopolysaccharides (LPSs), monocytes and macrophages induce reactive oxygen species (ROS) and cytokines, leading to the development of oxidative stress, apoptosis, and inflammatory responses in all types of tissues [3]. Inflammatory responses are one of the body’s defense mechanisms against injury [4], and this can be caused by several factors, such as chemical exposure, physical injury, and pathogen infections. Among them, inflammation caused by bacterial infections upregulates the cytokines, such as IL-1β, IL-6, IL-8, and TNF-α. Because inflammation can initiate autoimmune diseases and accelerate cancers, regulating inflammation is important [5].

LPS, also known as an endotoxin, exists on the outer membrane of Gram-negative bacterial cell walls. Endotoxins interact with host cells and trigger immune responses [6]. LPS is typically composed of a hydrophobic domain of lipid A, non-repeating oligosaccharide core, and an O-antigen, a distal chain which is composed of repeating saccharide subunits. LPS significantly stimulates the innate immune systems, and LPS-mediated signaling can result in a variety of pathologies. Septic shock is one of these, and it can result in severe cases [7].

Toll-like receptors (TLRs) recognize pathogen-associated molecular patterns (PAMPs), such as LPS, single-strand RNA, and flagellin. Among the TLR family, TLR4 recognizes LPS with the highly conserved lipid A portion serving as an important signature for receptor complex recognition [8,9]. Small amounts of LPS released by bacteria can trigger powerful innate immune responses. LPS is recognized by LPS transfer cascade proteins, including LPS binding protein (LBP), cluster of differentiation 14 (CD14), myeloid differentiation factor 2 (MD2), and TLR4 [10]. First, LPS micelles, which are formed when the LPS concentration is higher than the critical concentration, bind LBP. Through electrostatic interactions between LBP and CD14, LPS is transferred to CD14. By interacting with the TLR4-LRR15 domain, LPS is transferred from CD14 to MD2 [7]. LPS binding to the MD2/TLR4 complex leads to dimerization, forming (MD2/TLR4/LPS)_2_ homodimers. This aggregation initiates intracellular signaling and activates transcription factors, such as nuclear factor-kappa B (NF-κB) and interferon response factor 3 (IRF3), which produce pro-inflammatory cytokines [9,11]. 

LPS rapidly activates NF-κB and c-Jun N-terminal kinase (JNK) signaling pathways in THP-1 monocytes [12]. These pathways activate various transcription factors such as NF-κB (p50 and p65) and AP-1 (c-Fos and c-Jun), which modulate the induction of pro-inflammatory cytokines (TNFα and IL-1) and inflammatory mediators [13]. Because TLR4 and NF-κB/AP-1 signaling cascades play major roles in inflammatory responses caused by the infection of PAMPs such as LPS, regulating TLR4 and NF-κB/AP-1 is an attractive target for both acute and chronic inflammatory diseases [14,15,16].

(E)-2-methoxy-4-[3-(4-methoxyphenyl) prop-1-en-1-yl] phenol (MMPP) is a novel synthetic analog derived from(E)-2,4-bis(p-hydroxyphenyl)-2-butenal (BHPB). BHPB is a Maillard reaction product synthesized from fructose–tyrosine and has been shown to have an anti-inflammatory effect. However, BHPB has the fatal disadvantage of being structurally unstable and difficult to use as a drug [17,18]. For this reason, MMPP, which is more stable than BHPB, was recently synthesized. MMPP can inhibit the inflammatory response induced by the NF-κB pathway by binding more strongly to the ATP binding site of IKKβ [18,19]. Therefore, MMPP, which exhibits less toxicity, is a promising candidate drug, as it can function as a nonsteroidal anti-inflammatory drug (NSAID). Previous studies have revealed that MMPP exerts anti-inflammatory and anticancer effects by downregulating the STAT3 pathway in several cell lines, such as ovarian cancer cells, cervical cancer cells, and non-small lung cancer cells [20,21,22]. It has also been reported that MMPP may be a potential drug for metabolic disorders and type 2 diabetes, as it can increase insulin sensitivity as a PPARγ agonist [23]. However, it remains unclear whether MMPP acts as an antagonist of MD2 and inhibits the MD2/TLR4 pathway. Therefore, in this study, we aimed to evaluate the anti-inflammatory effects and the underlying molecular mechanisms of action in LPS-stimulated THP-1 monocytes. These findings provide insights into the role of candidate MD2 inhibitor targeting TLR4.

## 2. Results

### 2.1. Effects of MMPP and LPS on the Viability of THP-1 Cells

**Figure 1:**
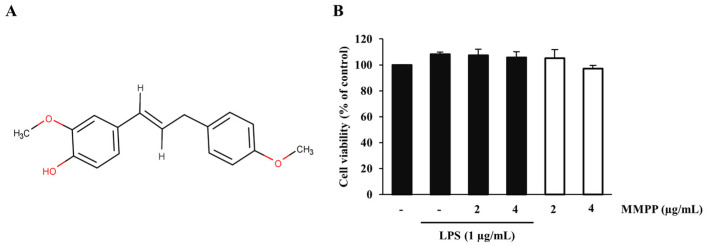
Chemical structure and cytotoxic effects of MMPP. (**A**) Chemical structure of MMPP. (**B**) The cytotoxic effects of MMPP and LPS on THP-1 cells were assessed by cell viability assay. THP-1 cells were co-treated with MMPP (2 and 4 µg/mL) and LPS (1 µg/mL) or MMPP alone for 24 h. Data are presented as the mean ± SD (*n* = 3).

The cytotoxicity of MMPP was assessed in the human monocyte-like cell line THP-1 by using a cell viability assay. THP-1 cells were co-treated with MMPP (2 and 4 µg/mL) and LPS (1 µg/mL) or MMPP alone for 24 h. The significant effect of MMPP on cell viability was not observed at any treated concentrations. (Figure 1B). MMPP (2 and 4 µg/mL) does not affect the cell viability of THP-1. 

### 2.2. Effects of MMPP on the mRNA Expression Levels of Pro-Inflammatory Cytokines in LPS-Treated THP-1 Cells

**Figure 2:**
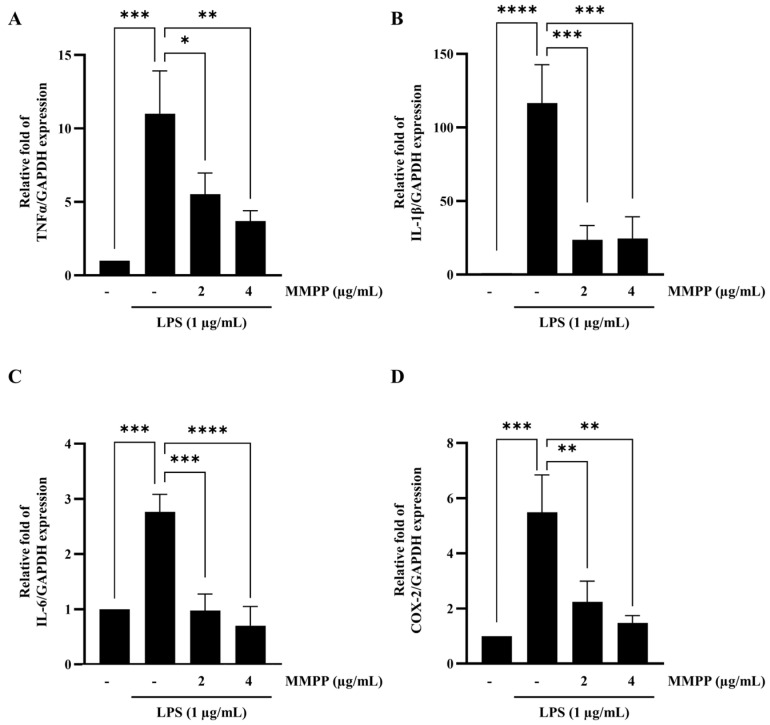
Inhibitory effects of MMPP on the expression of pro-inflammatory genes in LPS-stimulated THP-1 cells. (**A**–**D**) THP-1 cells were pretreated with MMPP (2 and 4 µg/mL) for 1 h and treated with LPS (1 µg/mL) for 2 h. The mRNA levels of TNF-α, IL-1β, IL-6, and COX-2 were determined by RT-qPCR. Data are presented as the mean ± SD (*n* = 3); * p < 0.05, ** p < 0.01, *** p < 0.001, and **** p < 0.0001 by one-way ANOVA.

To determine whether MMPP affects the pro-inflammatory cytokines increased by LPS, gene expression levels were analyzed by RT-qPCR. MMPP significantly reduced the expression of pro-inflammatory genes such as TNF-α, IL-1β, and IL-6, as well as an inflammatory mediator, COX-2, in a dose-dependent manner in LPS-stimulated THP-1 cells (Figure 2). MMPP exerts anti-inflammatory effects by downregulating the expression of inflammatory genes induced by LPS.

### 2.3. Effects of MMPP on the Phosphorylation of IKKα/β, IκBα, and the Nuclear Translocation of NF-κB in LPS-Treated THP-1 Cells

**Figure 3:**
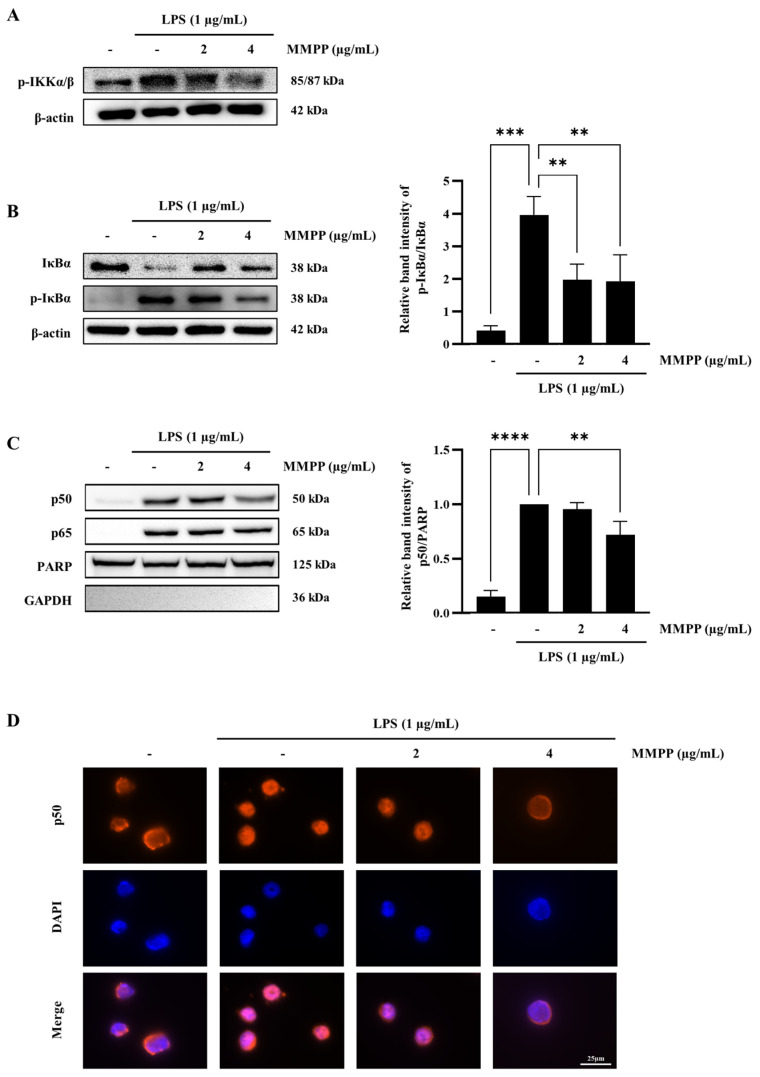
Inhibitory effects of MMPP on the expression of p-IKKα/β, p-IκBα/IκBα, NF-κB, and MAPKs in LPS-stimulated THP-1 cells. THP-1 cells were pretreated with MMPP (2 and 4 µg/mL) for 1 h and treated with LPS (1 µg/mL) for 30 min (**A**), 45 min (**B**), and 90 min (**C**,**D**). (**A**) The p-IKKα/β levels were evaluated by Western blot. (**B**) The p-IκBα and IκBα levels were evaluated by Western blot; p-IκBα to IκBα were evaluated by ImageJ software. Data are presented as the mean ± SD (*n* = 3); ** *p* < 0.01, and *** *p* < 0.001 by one-way ANOVA. (**C**) The nuclear translocations of p50 and p65 were evaluated by Western blot. PARP and GAPDH were used as positive and negative controls, respectively, for the nuclear and cytosolic fractions. The relative band intensities of p50 to PARP were evaluated by ImageJ software. Data are presented as the mean ± SD (*n* = 3); ** *p* < 0.01, and **** *p* < 0.0001 by one-way ANOVA. (**D**) The nuclear translocation of p50 was evaluated by immunofluorescence staining; p50 was labeled with Cy3 (red) in cells, and nuclei were labeled with DAPI (blue). (*n* = 3) Scale bar = 25 μm.

To determine whether MMPP regulated the phosphorylation of IKKα/β and IκB, the p-IKKα/β, IκB, and p-IκB levels were estimated by employing the Western blot. IKK and IκB play a role in the phosphorylation of IκB and inhibiting κB, respectively [15]. MMPP decreased the level of p-IKKα/β in a dose-dependent manner (Figure 3A). MMPP inhibited the phosphorylation and degradation of IκBα (Figure 3B). To confirm the nuclear translocation of NF-κB subunits such as p50 and p65, the cytosolic and nuclear fractions were separated. Both the p50 and p65 levels in the nuclear fraction were increased by LPS, but only p50 was decreased by MMPP in a dose-dependent manner (Figure 3C). The immunofluorescence staining also revealed that LPS induced the translocation of NF-κB p50 into the nucleus was attenuated by MMPP (Figure 3D). These results demonstrate that MMPP blocks the nuclear translocation of NF-κB p50 by regulating the phosphorylation of IKKα/β and IκBα.

### 2.4. Effects of MMPP on the Phosphorylation MAPKs and the Nuclear Translocation of AP-1 in LPS-Treated THP-1 Cells 

**Figure 4:**
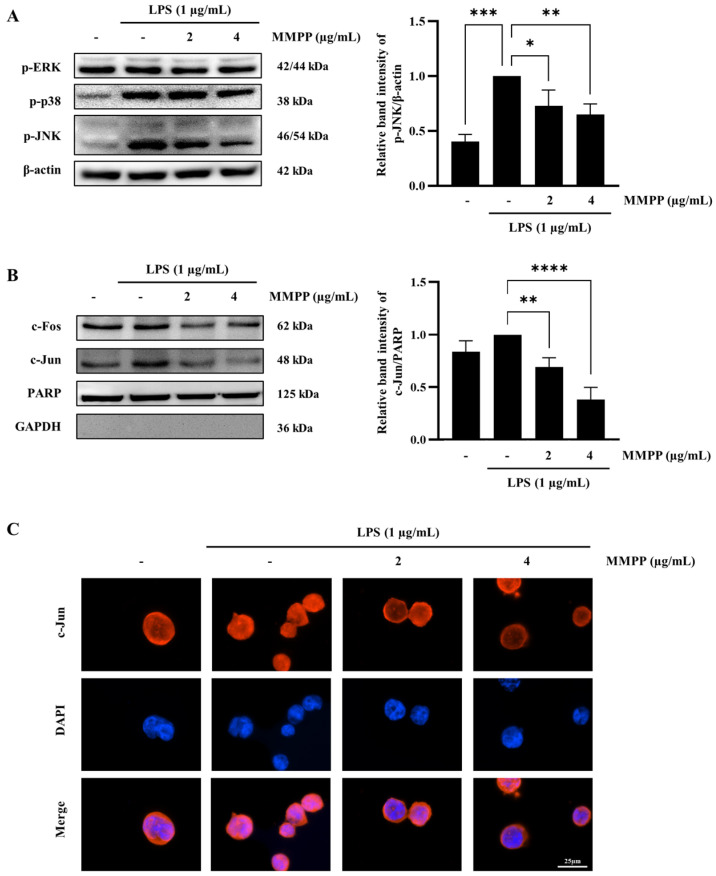
Inhibitory effects of MMPP on the expression of MAPKs and AP-1 in LPS-stimulated THP-1 cells. THP-1 cells were pretreated with MMPP (2 and 4 µg/mL) for 1 h and treated with LPS (1 µg/mL) for 60 min (**A**) and 90 min (**B**,**C**). (**A**) The phosphorylation levels of MAPKs were evaluated by Western blot. The relative band intensities of p-JNK to β-actin were evaluated by ImageJ software. Data are presented as the mean ± SD (*n* = 3); * p < 0.05, ** p < 0.01, and *** p < 0.001 by one-way ANOVA. (**B**) The nuclear translocations of c-Fos and c-Jun were evaluated by Western blot. PARP and GAPDH were used as positive and negative controls, respectively, for the nuclear and cytosolic fractions. The relative band intensities of c-Jun to PARP were evaluated by ImageJ software. Data are presented as the mean ± SD (*n* = 3); ** p < 0.01, and **** p < 0.0001 by one-way ANOVA. (**C**) The nuclear translocation of c-Jun was evaluated by immunofluorescence staining, c-Jun was labeled with Cy3 (red) in cells, and nuclei were labeled with DAPI (blue). Scale bar = 25 μm.

To determine the effect of MMPP on LPS-induced mitogen-activated protein kinase (MAPK), the levels of p-ERK, p-p38, and p-JNK were analyzed. The level of p-ERK was not altered by LPS or MMPP treatment. However, the p-p38 and p-JNK levels were increased by LPS, and p-p38 was marginally decreased only at a high MMPP concentration. The levels of p-JNK were significantly reduced in a dose-dependent manner (Figure 4A). The cytosolic and nuclear fractions were separated to confirm the nuclear translocation of c-Fos and c-Jun, but only the nuclear translocation of c-Jun was reduced by MMPP (Figure 4B). The immunofluorescence staining analysis also revealed that the nuclear translocation of c-Jun was suppressed by MMPP (Figure 4C). Taken together, the results indicate that MMPP reduced the translocation of c-Jun by inhibiting the phosphorylation of JNK.

### 2.5. Inhibitory Effects of MMPP on LPS Binding to CD14 and MD2 

**Figure 5:**
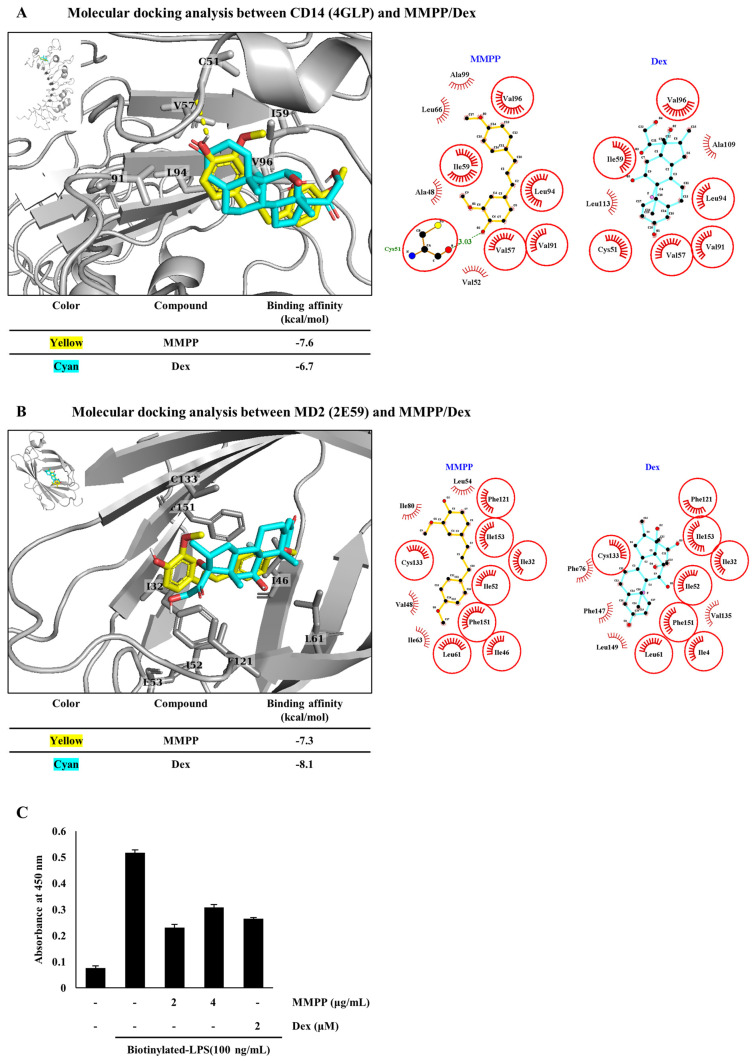
Inhibitory effects of MMPP on LPS binding to CD14 and MD2. (**A**,**B**) Molecular docking analysis showing the interactions between MMPP/Dex and CD14 or MD2. The PyMOL 3D diagram shows favorable docking results. The LigPlot+ 2D diagrams show the potential intermolecular interactions between MMPP (CID: 122517441)/Dex (CID: 5743) and CD14 (PDB code: 4GLP) (**A**) or between MMPP/Dex and MD2 (PDB code: 2E59) (**B**). MMPP is shown in yellow, and Dex in cyan. All atoms are voluntarily not shown. In the figures on the left side, only the residues of proteins in which the two ligands interact equally were shown. In the figures on the right side, the broken green lines represent the hydrogen bonds between target proteins and ligands. The red eyelash-like icons indicate the hydrophobic interactions between target proteins and ligands. In addition, the red circles indicate the residues where the two ligands interact equally. (**C**) LPS binding to MD2 was evaluated by an in vitro binding assay using recombinant MD2 (rMD2) and biotinylated LPS; rMD2 was coated in 96-well plates and blocked using 5% BSA in PBS; and MMPP or Dex was pre-incubated, followed by the addition of biotinylated LPS (100 ng/mL). The optical density was measured at 450 nm. The data represent one of the duplicates and are shown as the mean ± SD.

To simulate whether MMPP and Dex interfere with LPS binding to CD14 and MD2, we performed molecular docking analyses by using PyMOL, PyRx, and LigPlot+. As previously reported, Dex, which has recently been used for the treatment of allergies, autoimmune diseases, and even COVID-19, was used as a positive control [24,25]. MMPP was shown to have hydrophobic interactions with amino acid residues, such as Ala48, Val52, Val57, Ile59, Leu66, Val91, Val96, and Ala99, and it had a hydrogen bond in Cys51 to bind to CD14. MMPP and Dex were directly bound to CD14, with binding affinities of −7.6 kcal/mol and −6.7 kcal/mol, respectively (Figure 5A). MMPP also had hydrophobic interactions with amino acid residues such as Ile32, Ile46, Val48, Ile52, Leu54, Leu61, Ile63, Ile80, Phe121, Cys133, Phe151, and Ile153 to bind to the hydrophobic pocket of MD2. MMPP and Dex were bound to MD2, with high binding affinities of −7.3 kcal/mol and −8.0 kcal/mol, respectively (Figure 5B). These simulation results suggested that MMPP and Dex interfere with the binding of LPS to CD14 and MD2.

To determine whether MMPP inhibits the interaction between LPS and MD2, the level of LPS bound to MD2 was determined using an in vitro binding assay. The in vitro binding assay revealed that MMPP inhibited the association of recombinant MD2 (rMD2) and biotinylated LPS (Figure 5C). Considered together, these results suggest that MMPP competes with LPS and binds to CD14 and MD2.

## 3. Discussion

There are 13 subspecies of receptors in the mammalian TLR family, of which TLR4 is one of the most active. TLR4 mediates inflammatory responses by inducing the production of pro-inflammatory genes related to septic shock, acute lung injury, and cardiovascular disease [26]. Identifying effective methods for the treatment of excessive inflammatory responses is essential for human health. In this study, we confirmed the anti-inflammatory effects and underlying molecular mechanisms of MMPP in LPS-stimulated THP-1 monocytes. 

Before verifying the effectiveness of MMPP, we first determined whether 1 µg/mL of LPS and various concentrations of MMPP affected the cell viability. The results of the assay revealed that cytotoxicity was not observed at any concentration of MMPP (2 and 4 µg/mL) or when LPS was co-treated with MMPP (Figure 1B). Thus, we performed experiments with specified concentrations of MMPP (2 and 4 µg/mL) and LPS (1 µg/mL). 

TLR4 is expressed in the immune cells of myeloid origin, such as monocytes, dendritic cells, and macrophages [27]. Most myeloid cells express the pattern-recognition receptor CD14 on the plasma membrane, which enhances innate immune responses [28]. LPS-induced TLR4 stimulates the NF-kB and AP-1 pathways, which release pro-inflammatory mediators and cytokines and mediators such as TNFα, IL-1β, IL-6,COX-2, and iNOS [29,30]. Accordingly, we investigated the regulatory effect of MMPP on pro-inflammatory genes in LPS-stimulated THP-1 monocytes. We confirmed that MMPP downregulated the expression levels of pro-inflammatory cytokines such as TNFα, IL-1β, and IL-6 and the pro-inflammatory mediator COX-2 in a dose-dependent manner (Figure 2). MMPP inhibits the inflammatory effects of LPS in THP-1 cells.

LPS binding to TLR4 activates the canonical NF-κBs pathway, and IKKβ in the IKK complex is phosphorylated. The transcription factor NF-κB family, including p65 (RelA), RelB, c-Rel, p52, and p50, has crucial roles in innate immune response, inflammation, and cancer initiation and progression [15]. To confirm whether MMPP can suppress the NF-κB pathway, we sequentially identified whether IKK, IκBα, and NF-κB (p50 and p65) are activated. MMPP downregulated LPS-induced IKK phosphorylation in a dose-dependent manner (Figure 3A). MMPP has the ability to bind to the ATP binding site of IKKβ and blocks the phosphorylation of downstream components such as IκBα [18]. As expected, the phosphorylation level of IκBa was reduced by MMPP (Figure 3B). The phosphorylated IκBα undergoes polyubiquitylation and proteasomal degradation [8]. The IκBα level was markedly decreased under LPS treatment alone (Figure 3B), resulting in less accessible NF-κB, which prevents it from translocating into the nucleus. MMPP inhibited the nuclear translocation of p50 in a dose-dependent manner (Figure 3C,D). These results demonstrate that MMPP reduces the IKK/IκBα inflammatory signaling pathway and inhibits p50 translocation from the cytosol to the nucleus, suppressing the expression of pro-inflammatory mediators and cytokines in LPS-stimulated THP-1 cells. Thus, MMPP exhibits anti-inflammatory activity by blocking the NF-κB pathway by binding to the ATP binding site of IKKβ.

MAPKs, which are found in all the eukaryotic cells, include ERK, p38, and JNK. They regulate various biological processes, such as inflammatory response, cell proliferation, differentiation, and apoptosis [16,31]. Among MAPKs, JNK is also known as stress-activated protein kinase (SAPK) because of its strong activation under cellular stress induced by bacterial toxins such as LPS. The JNK signaling pathway can be activated by LPS recognizing cell surface proteins such as CD14, CD36, MD2, and TLR4 [31]. Thus, we investigated whether the phosphorylation level of MAPKs was increased by LPS and whether this activation was ameliorated by MMPP. MMPP effectively reduced the LPS-induced JNK phosphorylation (Figure 4A) and inhibited the nuclear translocation of c-Jun (Figure 4B,C). Thus, MMPP reduced the LPS-induced inflammatory response by inhibiting the JNK/AP-1 signaling pathways. By downregulating the phosphorylation of JNK, MMPP can play an important role in many biological diseases by regulating various cellular processes, including inflammatory response, cell death, and cell survival.

During the inflammatory response to LPS, endotoxins form micelles that first bind to LBP, and LPS is transmitted to CD14. Subsequently, CD14 shifts LPS to the MD2/TLR4 complex, followed by dimerization of the MD2/TLR4/LPS complex, forming (MD2/TLR4/LPS)_2_. The pro-inflammatory signaling pathway, which is involved in the innate immune response, is then activated [26,27]. Therefore, we hypothesized that MMPP would bind to CD14 and MD2 to suppress the LPS binding, thereby suppressing downstream signaling. Dex was used as the positive control. It is a synthetic steroid that has been used for years in clinical trials because of its anti-allergic, anti-inflammatory, and immunosuppressive properties [32]. First, we confirmed ligand–protein bindings between MMPP (or Dex) and CD14 or between MMPP (or Dex) and MD2 were performed using PyMOL, PyRx, and LigPlot+. According to the description of the PyRx server, the more negative the value for the binding affinity, the better the predicted binding between the ligand and macromolecule [15]. On the basis, the results on the molecular docking analyses demonstrated that MMPP and Dex are directly bound to CD14 and MD2 (Figure 5A,B). Lastly, we also revealed that MMPP prevented LPS from binding to MD2 by using in vitro binding assay (Figure 5C). These results indicate that MMPP interferes with the binding of LPS to CD14 and MD2 on the cell membrane surface and modulates the TLR4-mediated inflammatory signaling pathway in LPS-stimulated THP-1 cells. 

In summary, the results of this study show that MMPP (a) blocks the nuclear translocation of NF-κB p50 by regulating the phosphorylation of IKKα/β and IκBα, (b) reduces the translocation of c-Jun by inhibiting the phosphorylation of JNK, (c) competes with LPS in binding to CD14 and MD2, and (d) inhibits the NF-κB and JNK pathways by binding to MD2 in LPS-stimulated THP-1 cells (in vitro). Furthermore, MMPP (at 2 or 4 μg/mL) is not toxic to THP-1 cells.

In a previous study, the ADME properties of MMPP were already analyzed [21]. The ADME analyses revealed that MMPP is less toxic than BHPB. The in vivo effect of MMPP was not confirmed in this study. In further studies, the in vivo anti-inflammatory effect of MMPP will be investigated. The in vivo anti-inflammatory testing (relative to an appropriate standard reference/drug) may enhance the impact of the in vitro effect of the MMPP study.

## 4. Materials and Methods

### 4.1. Reagents

MMPP, produced as previously reported [20], was kindly donated by Prof. Hong JT (Chungbuk National University, Cheongju, Republic of Korea). Dexamethasone (Dex) and LPS from *Escherichia coli O111:B4* (L2630) were purchased from Sigma-Aldrich (St. Louis, MO, USA). 

### 4.2. Cell Culture

The human monocyte-like cell line THP-1 was obtained from the American Type Culture Collection (ATCC) (Manassas, VA, USA). The THP-1 cells were incubated at 37 °C and 5% CO_2_ in Roswell Park Memorial Institute (RPMI) cell culture medium (Welgene Incorporation, Daegu, Korea) supplemented with 10% (*v*/*v*) fetal bovine serum (FBS) (Hyclone Laboratories, Logan, UT, USA), penicillin (100 U/mL), and streptomycin (100 μg/mL). Prior to use, the FBS was thermally inactivated at 56 °C for 30 min.

### 4.3. Cell Viability Assay

Cell viability was estimated using the 3-(4,5-dimethylthiazol-2-yl)-5-(3-carboxymethoxy phenyl)-2-(4-sulfophenyl)-2H-tetrazolium (MTS) assay. The CellTiter 96 Aqueous One Solution Assay (Promega, Madison, WI, USA) was used. THP-1 cells (3 × 10^4^ cells/well) were seeded in 96-well plates in 100 μL of the medium. Moreover, the cells were co-treated with MMPP (2 and 4 µg/mL) and LPS (1 µg/mL), or MMPP (2 and 4 µg/mL) alone for 24 h. A 20 µL of the reagent was then added to each well, and the mixture was incubated for another 30 min. Absorbance at 492 nm was measured using a microplate reader (Apollo LB 9110; Berthold Technologies GmbH, Bad Wildbad, Germany). 

### 4.4. RNA Isolation and Quantitative Real-Time PCR (RT-qPCR)

THP-1 cells were seeded in 6-well plates (4.0 × 10^5^ cells/well). The cells were then pretreated for 1 h with MMPP (2 and 4 µg/mL) and then treated for another 2 h with LPS (1 µg/mL). According to the manufacturer’s instruction, the cells were collected and lysed using the easy-BLUE™ Total RNA Extraction Kit (iNtRon Biotechnology, Seoul, Republic of Korea) to isolate RNA. It was reverse-transcribed using M-MuLV reverse transcriptase (New England Biolabs, Ipswich, MA, USA) to synthesize complementary DAN (cDNA). RT-qPCR was conducted using TB Green Premix Ex Taq^TM^ (Takara Shuzo, Kyoto, Japan). A thermal cycler (Thermal Cycler Dicer Real-Time system; Takara Shuzo, Kyoto, Japan) was used to monitor the fluorescence accumulation of the PCR product. Using the comparative Ct method, the expression levels of the target genes were normalized to GAPDH. The number of cycles required for the PCR signal to exceed the threshold level was defined as Ct. Fold changes in the test group compared to the control group were calculated to be 2^−ΔΔCt^, where ΔΔCt = ΔCt_(test)_ − ΔCt_(control)_ = (Ct_(gene)_ − Ct_(reference)_)_test_ − (Ct_(target gene)_ − Ct_(reference)_)_control_ [33]. The primer sets used for qPCR are listed in Table 1.

### 4.5. Western Blot

THP-1 cells were seeded in 6-well plates (4.0 × 10^5^ cells/well). The cells were then pretreated for 1 h with MMPP (2 and 4 µg/mL) before treatment with LPS (1 µg/mL). Harvested cells were washed with phosphate-buffered saline (PBS) and lysed in RIPA buffer containing 150 mM NaCl, 0.1% sodium dodecyl sulfate, 0.25% sodium deoxycholate, 1 mM ethylene diamine tetra-acetic acid, 1% NP40, 0.4 mM phenylmethylsulfonyl fluoride, 1 mM orthovanadate, 1 mM ethylene glycol tetra-acetic acid, 50 mM Tris (pH 7.4), and aprotinin (10 µg/mL) at 4 °C for 30 min. The 9% sodium dodecyl sulfate–polyacrylamide gel electrophoresis (SDS-PAGE) was used to separate equal amounts of quantified proteins (25 µg). The proteins separated by size were transferred onto a polyvinylidene difluoride (PVDF) membrane. The PVDF membrane was blocked with 5% skim milk at room temperature for 1 h. The specific primary antibodies (1:1000 dilution) used were as follows: p-p38 (#sc-17852-R), p50 (#sc-8414), c-Fos (#sc-52), c-Jun (#sc-45), GAPDH (#sc-477244), β-actin (#sc-47778) (Santa Cruz Biotechnology, Dallas, TX, USA), p-ERK (#9101S), p-JNK (#9251S), IKKα/β (#2697T), IκBα (#4812S), p- IκBα (#2859S), p65 (#4764S), and PARP (#9542S) (Cell Signaling Technology, Danvers, MA, USA). Horseradish peroxidase (HRP) conjugated mouse-IgGκ light chain binding protein (1:3000 dilution) or HRP conjugated goat anti-rabbit IgG-heavy and light chain antibody (1:5000 dilution) was used to detect the target primary antibody. An enhanced chemiluminescence (ECL) WesternBright™ Western blot detection kit (Advansta, CA, USA) was used to visualize protein bands. Densitometric graphs of three independent experiments were generated using ImageJ software (version 1.5) [34]. β-actin was used to normalize the band intensities of the target proteins.

### 4.6. Separation of Cytosolic and Nuclear Fractions

THP-1 cells were seeded in 6-well plates (4.0 × 10^5^ cells/well) and pretreated for 1 h with MMPP (2 and 4 µg/mL) and then treated for another 1.5 h with LPS (1 µg/mL). According to the manufacturer’s instructions, cells attached to the bottom of the 6-well plates were harvested by using trypsin-EDTA. To prepare cytosolic and nuclear fractions from the collected cells, NE-PER Nuclear and Cytoplasmic Extraction Reagents (Thermo Fisher Scientific Inc., Waltham, MA, USA) were used. Equal quantities of proteins from these extracts were analyzed by Western blot.

### 4.7. Immunofluorescence Staining

An 8-well cell culture slide was coated with poly-L-lysine (0.01% solution; Sigma, St. Louis, MO, USA). THP-1 cells (4.0 × 10^5^ cells/well) were seeded and pretreated for 1 h with MMPP (2 and 4 µg/mL) and then treated for another 1.5 h with LPS (1 µg/mL). A 200 μL of 4% paraformaldehyde (PFA) was incubated at room temperature for 10 min for the cell fixation. After washing twice with PBS, 200 μL of 100% methanol was incubated at 4 °C for 10 min for cell permeabilization. Then 1% BSA in PBS was used to block the nonspecific binding of antibodies. Specific antibodies against p50 and c-Jun (1:200 dilution) were incubated overnight at 4 °C. Normal rabbit IgG was used as a control at the same concentration of primary antibody. The cells were then incubated with goat anti-rabbit IgG secondary antibody labeled with Cy3 (Merck Millipore, Darmstadt, Germany, 1:400 dilution) for 1 h. The cells were stained with 4,6-diamidino-2-phenylindole (DAPI) (1:1000 dilution) (Sigma, St. Louis, MO, USA). A confocal fluorescence microscope (EVOSTM M7000; Thermo Fisher Scientific Inc., Waltham, MA, USA) with a 100× objective was used to obtain the fluorescence images.

### 4.8. Molecular Docking Analysis

The docking software PyRx v0.9.2, together with AutoDock Vina v1.2.0, was used for all docking analyses, as previously reported [35,36]. The grid box was centered on the target protein, and its size was adjusted to include the whole protein. The crystal structures of CD14 (PDB code: 4GLP) and MD2 (PDB code: 2E59) from the Protein Data Bank (PDB) and the ligand structures of MMPP (CID:122517441) and Dex (CID:5743) from PubChem were used for the docking analysis, as previously reported [24,37]. The docked complexes of the target proteins and ligands were visualized using the PyMOL molecular visualization system (version 2.5.4). The interactions between the target proteins and ligands were analyzed using the LigPlot+ 2D program (version 2.2), as previously reported [38]. The docking results are represented based on the binding affinity of the protein–ligand complex (kcal/mol).

### 4.9. In Vitro Assay for MMPP Binding to MD2 

The recombinant MD2 (rMD2, 1 μg/mL) (R&D Systems, Minneapolis, MN, USA) protein diluted in PBS was immobilized in 96-well plates (Thermo Fisher Scientific Inc., Waltham, MA, USA) and incubated at 37 °C for 2 h. The wells were washed with PBS, followed by blocking with 300 μL of 5% BSA in PBS at room temperature overnight. The plate was then washed with PBST (PBS with 0.05% Tween-20) [10]. MMPP and Dex were pre-incubated at 37 °C for 1 h. Then biotinylated LPS (100 ng/mL) (Invivogen, San Diego, CA, USA) was added and incubated at room temperature for 1 h. Streptavidin conjugated to horseradish peroxidase (HRP) was incubated at 37 °C for 1.5 h [37]. The activity of HRP was determined using KLP SureBlue^TM^ TMB substrate, and the reaction was stopped by 2.5 N H_2_SO_4_. The optical density of each well was measured at a wavelength of 450 nm, using a microplate reader (Apollo LB 9110; Berthold Technologies GmbH, Bad Wildbad, Germany).

### 4.10. Statistical Analysis

Data were obtained from three independent experiments and are presented as the mean ± standard deviation (SD). GraphPad Prism 9 software (GraphPad Software Inc., San Diego, CA, USA) was used for statistical analysis. Tukey’s honest significant difference test was used after a one-way analysis of variance (ANOVA). Statistical significance was set at * p < 0.05, ** p < 0.01, *** p < 0.001, and **** p < 0.0001 by one-way ANOVA.

## 5. Conclusions

**Figure 6:**
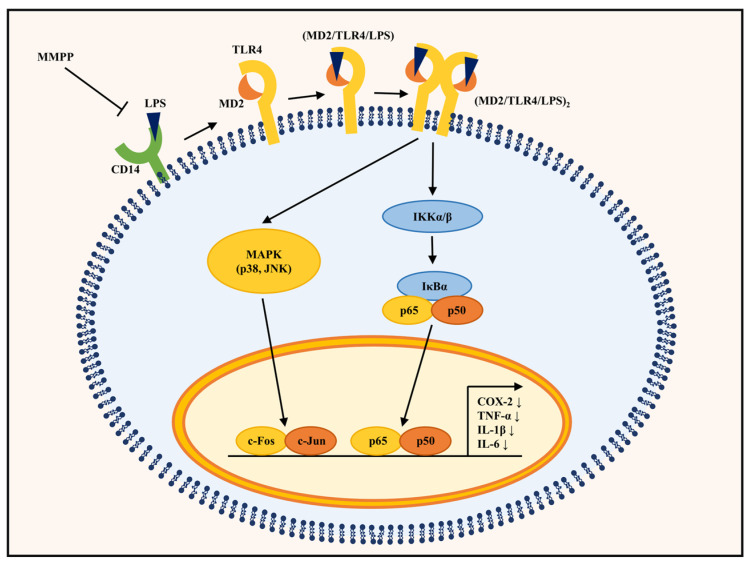
Schematic diagram of the effect of MMPP on the LPS-stimulated THP-1 cells. LPS binding to TLR4 is facilitated by CD14 and MD2. The binding of MMPP to CD14 and MD2 was revealed through molecular docking analysis and in vitro binding assay. MMPP treatment alleviated the phosphorylation of JNK and IKKαβ/IκBα and the nuclear translocation of NF-κB p50 and c-Jun, and it finally inhibited pro-inflammatory cytokines and inflammatory mediators such as TNF-α, IL-1β, IL-6, and COX-2.

Altogether, MMPP was found to inhibit the expression of pro-inflammatory cytokines such as TNF-α, IL-1β, and IL-6, as well as the inflammatory mediator COX-2, by binding to MD2, which is complexed with TLR4 in the plasma membrane. MMPP also downregulated the LPS-induced phosphorylation of IKK, IκB, and JNK and the nuclear translocation of NF-κB and AP-1 (Figure 6). Therefore, MMPP is a potent anti-inflammatory agent that targets MD2 directly.

## Figures and Tables

**Table 1:** Primer sequences of TNF-α, IL-1β, IL-6, COX-2, and GAPDH used in qPCR. The product sizes generated by each primer set are also shown.

Gene	Primer	Sequence (5′-3′)	Product Size (bp)
TNF-α	Forward	CTC TTC TGC CTG CTG CAC TTT G	175
	Reverse	ATG GGC TAC AGG CTT GTC ACT C	
IL-1β	Forward	CCA CAG ACC TTC CAG GAG AAT G	131
	Reverse	GTG CAG TTC AGT GAT CGT ACA GG	
IL-6	Forward	AGA CAG CCA CTC ACC TCT TCA G	132
	Reverse	TTC TGC CAG TGC CTC TTT GCT G	
COX-2	Forward	GAA ACC CAC TCC AAA CAC AG	156
	Reverse	CCC TCG CTT ATG ATC TGT CT	
GAPDH	Forward	AGA ACA TCA TCC CTG CCT CT	131
	Reverse	CTG CTT CAC CAC CTT CTT GA	

## Data Availability

Data are contained within the article.
